# Editorial: Long COVID and brain inflammation: unravelling mechanisms and potential therapies

**DOI:** 10.3389/fimmu.2025.1575669

**Published:** 2025-03-17

**Authors:** Fatemeh Saheb Sharif-Askari, Jacob Raber, Karem H. Alzoubi

**Affiliations:** ^1^ Research Institute for Medical and Health Science, University of Sharjah, Sharjah, United Arab Emirates; ^2^ Department of Pharmacy Practice and Pharmacotherapeutics, College of Pharmacy, University of Sharjah, Sharjah, United Arab Emirates; ^3^ Departments of Behavioral Neuroscience, Neurology, and Radiation Medicine, Division of Neuroscience, Oregon National Primate Research Center (ONPRC), Oregon Health & Science University, Portland, OR, United States

**Keywords:** long Covid, neuroinflammation, immune dysregulation, cognitive dysfunction, preclinical mice models

Patients with Long COVID often experience persistent brain-related symptoms, including brain fog, mood changes, and dizziness (1–3), likely driven by neuroinflammation even after the virus is cleared. Imaging studies have shown structural and functional changes that indicate ongoing inflammation in the Long COVID brain. The biological mechanisms underlying these symptoms are still not fully understood, and research continues to identify effective therapies to improve both the physical and mental health of those affected (2).

In this Research Topic, we explore research on Long COVID and its investigation through preclinical animal models.

**Figure 1:**
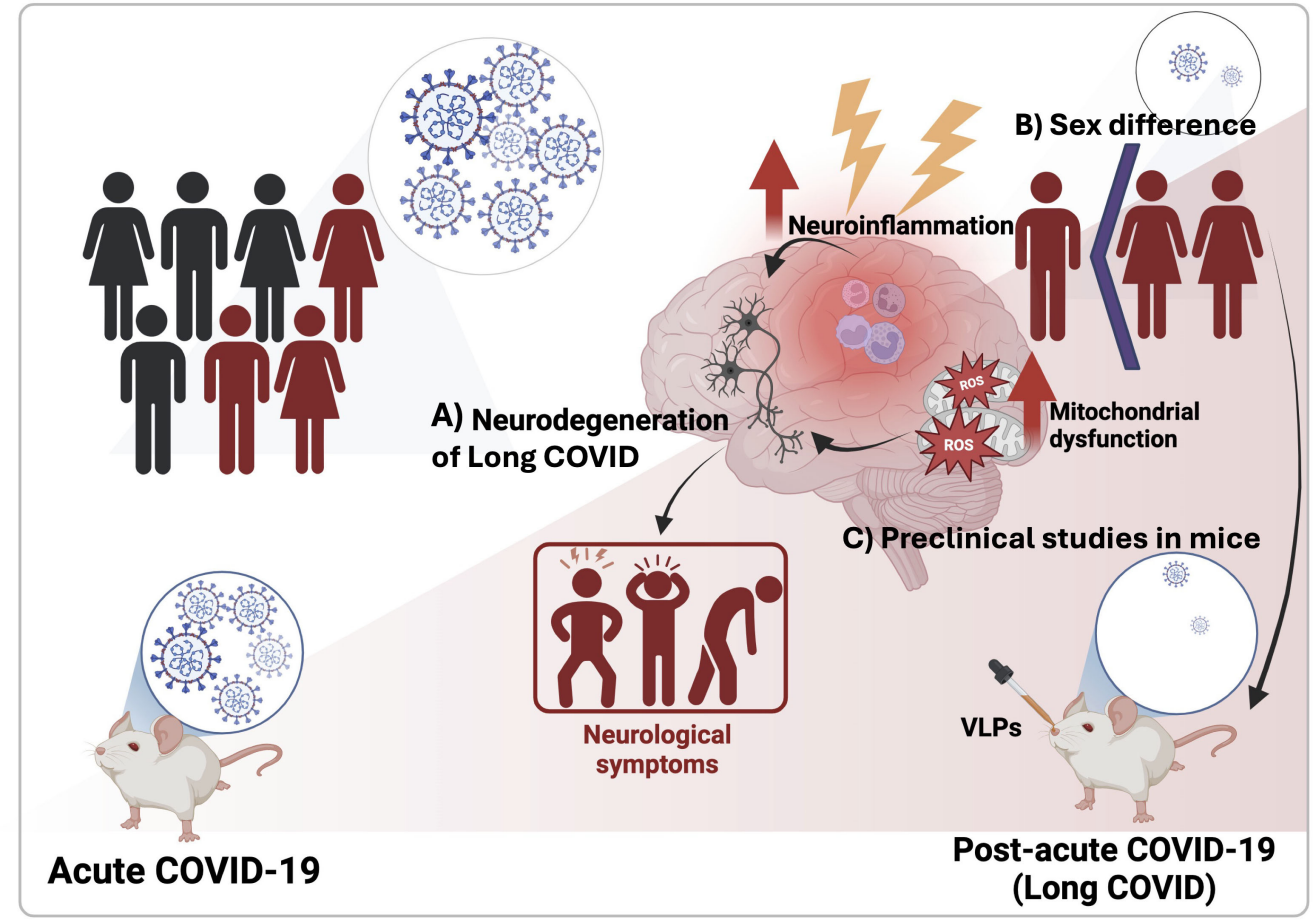
Neurological consequences of Long COVID. **(A)** In Long COVID, persistent neuroinflammation and mitochondrial dysfunction are implicated in ongoing neurodegeneration. **(B)** Certain populations, particularly females, are more susceptible due to hormonal and immune factors. **(C)** Preclinical models, including mice exposed to virus-like particles (VLPs), are being used to investigate the effects of Long COVID on the nervous system. These studies have linked neuroinflammation to behavioral disruptions, highlighting the need for further research into the neurological impacts of viral infections, particularly in Long COVID.

Missailidis et al. conducted RNA-Seq analysis of peripheral blood mononuclear cells (PBMCs) from individuals with Long COVID and those who had fully recovered COVID-19. Their findings revealed upregulation of ICOS and S1PR1, suggesting a persistent pro-inflammatory state (**Figure 1A**), as these genes are involved in immune cell survival and signaling. They also observed downregulation of LILRB1 and LILRB2, highlighting immune dysregulation as a distinguishing feature of Long COVID.

Lee et al. analyzed the expression of long noncoding RNAs (lncRNAs) in the brains of COVID-19 patients, identifying hundreds of differentially expressed lncRNAs compared to age- and sex-matched uninfected controls. Many of these lncRNAs correlated with cognitive decline and increased inflammation, aligning with cognitive dysfunction observed in Long COVID. These findings suggest a potential role for lncRNAs in the neurological effects of COVID-19, highlighting a key area for further research.

Noonong et al. proposed that mitochondrial dysfunction may play a crucial role in Long COVID, linking it to diabetes and oxidative stress (**Figure 1A**). Their hypothesis highlights mitochondria’s role in inflammation and metabolic homeostasis, suggesting broader systemic implications for viral recovery and chronic symptoms.

Hanafy and Jovin emphasized the role of chronic inflammation in exacerbating neurological symptoms commonly seen in patients with Long COVID, such as brain fog, extreme fatigue (asthenia), and depression- a condition they refer as “Brain FADE syndrome” (**Figure 1A**). They advocate for an integrated treatment approach that targets both inflammatory pathways and associated mental health challenges.

Gu et al. investigated sex differences in COVID-induced autoimmunity and neurological effects, highlighting how female sex hormones and X-chromosome factors may increase women’s susceptibility to Long COVID (**Figure 1B**). They proposed that COVID-19 may disturb immune tolerance, leading to autoantibodies infiltration into the central nervous system. They also suggested that COVID-19 could disrupt the female microbiome, contributing to neural damage, including demyelination, neuroinflammation, and neurodegeneration. Understanding these sex differences could help develop strategies to mitigate COVID-related neurological injuries.

Bustamante et al. explored risk factors for developing Long COVID, including a history of severe illness and intensive care. Using preclinical animal models, they examined the involvement of the nervous system in inflammation through the psychoneuroimmunoendocrine axes. They proposed that Long COVID involves peripheral and central sensitization, leading to dysregulation and chronic inflammation, and discussed therapeutic strategies to modulate these inflammation responses.

Dai et al. reviewed the utility of preclinical animal models in understanding Long COVID. They highlighted key features replicated in these models, including lung fibrosis, hyperglycemia, and neurological sequelae, while acknowledging limitations such as restricted genetic diversity and challenges in modeling Long COVID pathology. To improve translational relevance, they proposed incorporating genetically diverse populations, conducting longitudinal studies, and aligning animal findings with clinical data.

Singh et al. investigated the Long COVID implications of the Delta variant using K18-hACE2 mice. They found robust inflammatory responses linked to neuropsychiatric symptoms and motor behavior changes during acute infections, with persistent immune activation post-infection. Post-acute infection, the brain showed no detectable viral RNA and minimal residential immune cell activation in surviving mice. However, transcriptome analysis revealed persistent activation of immune pathways, including humoral responses, complement, phagocytosis, along with gene expression linked to ataxia telangiectasia, impaired cognitive function, and neuronal dysfunction. Surviving mice exhibited strong neutralizing antibodies against both Delta and Omicron variants, months after the infection.

O’Niel et al. used virus-like particles (VLPs) expressing SARS-CoV-2 structural proteins (nucleocapsid (N), membrane (M), envelope (E) and spike (S), in human apolipoprotein E (apoE)-targeted replacement mice (**Figure 1C**). The study found apoE isoform-dependent effects on behavioral measures, with E2 mice more affected than E3 or E4 mice, despite E2 being linked to a lower Alzheimer’s disease risk. VLPs also caused behavioral and circadian disruptions independent of apoE isoform, even in the absence of viral replication. Increased susceptibility in E2 mice was associated with elevated hippocampal CCL11, similar to CCL11 elevations seen in humans with cognitive symptoms after COVID-19 exposure. The authors emphasize the need for further research to better understand and treat neurological conditions associated with viral infections, especially as many continue to struggle with Long COVID.

The ongoing research discussed in this Research Topic reveals significant progress toward understanding the biological mechanisms of Long COVID. As we continue to uncover the complexities of the condition, further preclinical and clinical studies are critical for improving the well-being and brain function of those affected by Long COVID and other related neurological conditions.
